# Type 2 diabetes mellitus and incident carotid atherosclerosis: a retrospective cohort study on composite indices

**DOI:** 10.3389/fendo.2026.1904890

**Published:** 2026-09-03

**Authors:** Lei Li, Wufuer Aini, Sheng Jiang

**Affiliations:** Department of Endocrinology, The First Affiliated Hospital of Xinjiang Medical University, Urumqi, China

**Keywords:** carotid atherosclerosis, composite indices, retrospective cohort study, systemic immune-inflammation index, systemic inflammatory response index, type 2 diabetes mellitus

## Abstract

**Background:**

Type 2 diabetes mellitus (T2DM) is a major global health challenge, with atherosclerotic cardiovascular disease (ASCVD) representing a leading cause of mortality. Carotid atherosclerosis (CAS), an early manifestation of ASCVD, can be assessed non-invasively via carotid ultrasonography. Composite indices derived from routine laboratory tests may offer comprehensive assessment of metabolic and inflammatory status, but systematic comparisons for predicting incident CAS in T2DM are limited.

**Methods:**

This retrospective cohort study included 15,492 T2DM patients with ≥2 hospitalizations (2018–2024). Baseline was the first hospitalization; subsequent admissions served as follow-up. Seventeen composite indices were calculated from baseline laboratory data. Cox proportional-hazards regression with time-dependent coefficients was used as the primary analysis. Stratified and traditional Cox models were performed as sensitivity analyses. The Benjamini–Hochberg method was applied for multiple comparison correction.

**Results:**

Over a median follow-up of 306.5 days, 1,841 patients (11.88%) developed CAS. In fully adjusted models, higher quartile levels of SII (HR = 1.38, P = 0.0046), SIRI (HR = 1.37, P = 0.0061), NLR (HR = 1.40, P = 0.0028), and NHR (HR = 1.34, P = 0.0115) were significantly associated with increased CAS risk. Albumin showed an inverse association (HR = 0.74, P = 0.0102). All associations remained significant after FDR correction (Q < 0.05). Age- and sex-stratified analyses suggested potential heterogeneity, though interactions were not significant.

**Conclusions:**

SII, SIRI, NLR, and NHR were independently associated with increased CAS risk in T2DM patients, while albumin was protective. These inflammation-based composite indices may aid in CAS risk stratification. However, findings should be considered exploratory due to the retrospective design; prospective validation is warranted.

## 1 Introduction

Type 2 diabetes mellitus (T2DM) has emerged as a global public health challenge (1). According to the International Diabetes Federation, the number of people with diabetes exceeded 537 million in 2021, with T2DM accounting for over 90% of cases (2). The morbidity and mortality associated with T2DM are largely ascribable to vascular complications, particularly atherosclerotic cardiovascular disease (ASCVD) (3). Carotid atherosclerosis (CAS), an early manifestation and independent predictor of ASCVD, can be assessed non-invasively via carotid ultrasonography by measuring intima-media thickness (IMT) and plaque formation and is closely linked to the future risk of stroke and myocardial infarction (4). Therefore, the accurate identification of CAS-related risk factors in the high-risk T2DM population is of major clinical significance for early intervention and improved prognosis (5).

The pathophysiology of CAS in T2DM is complex, involving the interplay of hyperglycemic toxicity, insulin resistance, chronic low-grade inflammation, and lipid metabolism disorders (6–8). Traditional single risk indicators (e.g., blood glucose, lipids) may not fully capture the integrated effects of these complex pathological processes. In recent years, a range of composite indices have been developed, combining routine laboratory parameters to provide a multi-dimensional assessment of an individual’s metabolic, inflammatory, and immune status (9). The triglyceride-glucose (TyG) index has been proposed as a simple surrogate marker of insulin resistance (10). The Systemic Immune-Inflammation Index (SII) and the Systemic Inflammatory Response Index (SIRI) integrate platelet, neutrophil, lymphocyte, and monocyte counts to reflect the overall balance of the inflammatory network (11). Indices such as the neutrophil-to-high-density lipoprotein cholesterol ratio (NHR), monocyte-to-HDL-C ratio (MHR), lymphocyte-to-HDL-C ratio (LHR), and platelet-to-HDL-C ratio (PHR) combine inflammatory cell counts with the anti-inflammatory lipoprotein HDL-C, theoretically assessing the relative balance between pro-inflammatory and anti-inflammatory capacities, thus establishing a dual “attack-defense” evaluation framework (12).

While previous studies have investigated the association of some of these indices with vascular complications in T2DM, most have focused on one or a few indices, and the majority have been cross-sectional in design, limiting causal inference. This retrospective cohort study, including 15,492 T2DM patients, aimed to: (1) systematically compare the strength and direction of associations of 17 composite indices with incident CAS, and (2) explore the heterogeneity of these associations through age- and sex-stratified subgroup analyses, providing evidence-based support for risk stratification in T2DM.

## 2 Materials and methods

### 2.1 Study design and population

**Figure 1:**
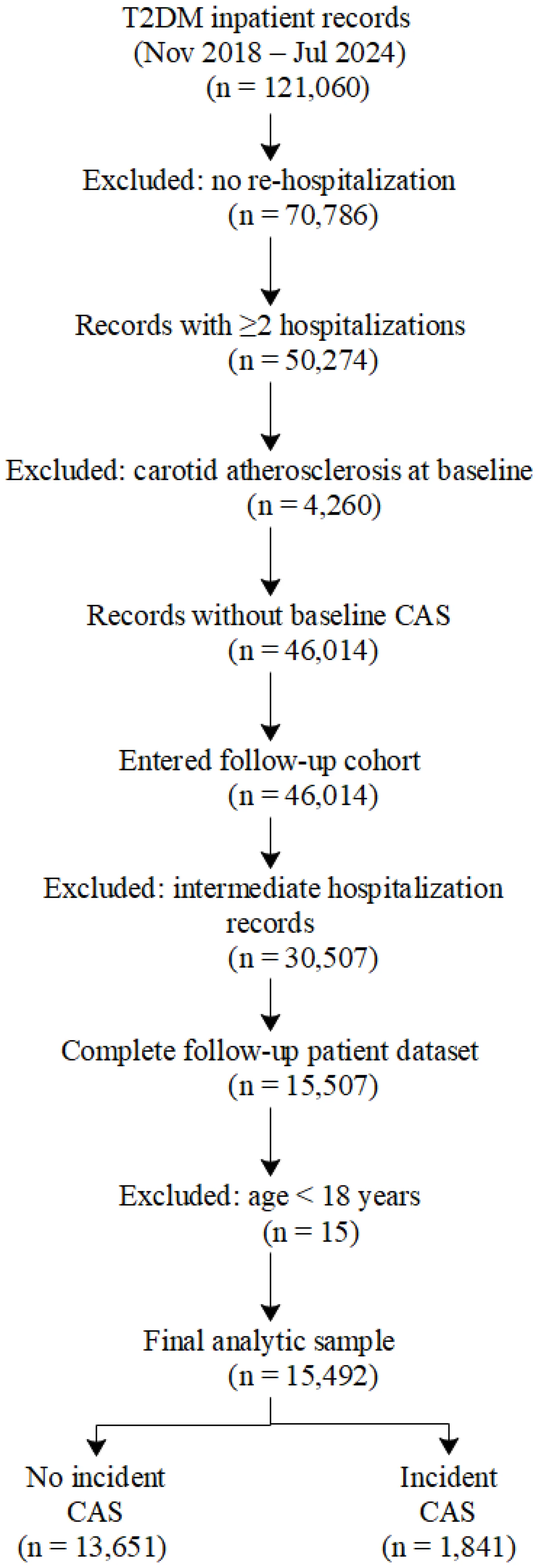
Flowchart of patient selection.

This was a single-center retrospective cohort study conducted at the First Affiliated Hospital of Xinjiang Medical University between November 2018 and July 2024. Hospitalization records of all patients with T2DM during the study period were extracted from the hospital’s electronic medical record (EMR) system. For patients with multiple hospitalizations, the first hospitalization was defined as the baseline visit, and subsequent hospitalizations were used for follow-up. Ultimately, 15,492 unique patients with at least two hospitalization records met the inclusion criteria. The unit of analysis was the individual patient rather than the hospitalization record. For patients with multiple admissions, the first admission defined baseline, and all subsequent admissions were used for longitudinal follow-up (**Figure 1**).

This retrospective study was approved by the Ethics Committee of the First Affiliated Hospital of Xinjiang Medical University (approval No. 20250926-06; 20251218–34) and was conducted in accordance with the Declaration of Helsinki. The need for informed consent was waived by the ethics committee because the study involved only de-identified data from routine clinical care and posed no more than minimal risk to patients.

### 2.2 Study participants

#### 2.2.1 Inclusion criteria

1. Hospitalized patients with a confirmed diagnosis of T2DM;

2. At least two complete hospitalization records;

3. Age ≥ 18 years.

#### 2.2.2 Exclusion criteria

1. Patients with a confirmed diagnosis of carotid atherosclerosis at baseline;

2. Patients without valid outcome assessment during follow-up.

### 2.3 Data collection and variable definitions

#### 2.3.1 Data extraction

Patient hospitalization records were extracted from the hospital EMR system, including: medical record number, admission number, admission date, discharge date, admission diagnosis, and all diagnosis fields. Records were chronologically ordered for each patient, with the first hospitalization defined as baseline and subsequent hospitalizations as follow-up.

#### 2.3.2 Definition and identification of carotid atherosclerosis

Carotid atherosclerosis (CAS) was identified through systematic retrieval of International Classification of Diseases (ICD) diagnosis fields from the electronic medical records. CAS diagnoses were confirmed by carotid ultrasonography and ascertained using ICD-9 and ICD-10 codes, supplemented by a keyword search of all diagnosis fields. The search terms included: “carotid atherosclerosis,” “carotid plaque,” “carotid sclerosis,” and “carotid stenosis” (with variations for anatomical sites including internal, common, and external carotid arteries). Patients with any diagnosis field containing these terms were classified as having CAS.

#### 2.3.3 Outcome definition

The primary outcome was incident CAS during follow-up, defined as:

##### 2.3.3.1 Baseline status

Patients without CAS based on the above diagnostic criteria at the first hospitalization;

##### 2.3.3.2 Incident event

The first appearance of a CAS diagnosis during subsequent follow-up hospitalizations;

##### 2.3.3.3 Event time

The interval (in days) from the baseline hospitalization date to the date of the first CAS diagnosis;

##### 2.3.3.4 Censoring

Patients who did not develop CAS during follow-up were censored at the date of their last follow-up visit.

#### 2.3.4 Covariate definitions

Based on previous literature and clinical relevance, the following covariates were included:

##### 2.3.4.1 Demographic characteristics

Sex (male/female).

##### 2.3.4.2 Chronic disease history (binary variables)

Hypertension, coronary artery disease (CAD), dyslipidemia, diabetic kidney disease (DKD), chronic kidney disease (CKD), and non-alcoholic fatty liver disease (NAFLD). These variables were extracted from the discharge diagnosis fields in the EMR system and coded as binary variables (present/absent).

### 2.4 Composite index formulas

Based on a literature review, 17 composite indices were included in this study, reflecting body mass status, renal function, nutritional status, uric acid metabolism, inflammatory status, and lipid metabolism. All composite indices were calculated using results from fasting venous blood samples collected at baseline hospitalization.

#### 2.4.1 Body mass and renal function indices

1. Body Mass Index (BMI) = Weight (kg)/Height (m)².

2. Estimated Glomerular Filtration Rate (eGFR): directly recorded as measured values (mL/min/1.73 m²).

#### 2.4.2 Nutritional and metabolic indices

3. Albumin: directly recorded as measured values (g/L).

4. Uric Acid: directly recorded as measured values (μmol/L).

#### 2.4.3 Inflammatory cell integration indices

5. Systemic Immune-Inflammation Index (SII) = Platelet count × Neutrophil count/Lymphocyte count.

6. Systemic Inflammatory Response Index (SIRI) = Neutrophil count × Monocyte count/Lymphocyte count.

7. Neutrophil-to-Lymphocyte Ratio (NLR) = Neutrophil count/Lymphocyte count.

8. Platelet-to-Lymphocyte Ratio (PLR) = Platelet count/Lymphocyte count.

#### 2.4.4 Inflammation-lipid ratio indices

9. Neutrophil-to-High-Density Lipoprotein Cholesterol Ratio (NHR) = Neutrophil count/HDL-C.

10. Monocyte-to-High-Density Lipoprotein Cholesterol Ratio (MHR) = Monocyte count/HDL-C.

#### 2.4.5 Lipid metabolism and insulin resistance indices

11. Atherogenic Index of Plasma (AIP) = log_10_ (Triglycerides/HDL-C).

12. Triglyceride-Glycated Hemoglobin Index (TyG HbA1c) = ln [TG (mmol/L) × HbA1c (%)/2].

#### 2.4.6 Traditional lipid ratios and combination indices

13. Castelli Risk Index I (CRI-I) = Total Cholesterol/HDL-C.

14. LDL/HDL Ratio = LDL-C/HDL-C.

15. Non-High-Density Lipoprotein Cholesterol (Non-HDL-C) = Total Cholesterol − HDL-C.

16. Atherogenic Index (AI) = (Total Cholesterol − HDL-C)/HDL-C.

17. Lipoprotein Combine Index (LCI) = Total Cholesterol × Triglycerides × LDL-C/HDL-C.

### 2.5 Handling of missing data

In this study, completeness was 100% for categorical variables such as baseline demographic characteristics and medical history. However, some laboratory parameters (e.g., triglycerides, HDL-C, fasting blood glucose, creatinine, uric acid) had missing data. Multiple imputation by chained equations (MICE) was used to handle missing values to minimize potential bias and improve statistical power.

Multiple imputation was performed using the mice package in R. The imputation model included all analytical variables (including outcomes and covariates) as predictors. Predictive mean matching (PMM) was used for imputing continuous missing variables to ensure imputed values fell within the original observed range. Five imputed datasets were generated, with 20 iterations per dataset. Convergence and the consistency of imputed versus observed distributions were assessed using trace plots, density plots, and strip plots. The results indicated good convergence and reasonable imputation distributions.

### 2.6 Statistical analysis

All statistical analyses were performed using R software.

#### 2.6.1 Data description and comparison

Non-normally distributed continuous variables were presented as median (interquartile range) [M (P_25_, P_75_)], with between-group comparisons using the Mann–Whitney U test. Categorical variables were presented as frequencies and percentages (n, %), with between-group comparisons using the chi-square (χ²) test.

#### 2.6.2 Grouping of composite indices

Each composite index was categorized into quartiles (Q1–Q4) based on its distribution in the overall population, with the first quartile (Q1) as the reference group.

#### 2.6.3 Survival analysis

The Kaplan–Meier method was used to estimate the cumulative incidence of new-onset CAS, and survival curves were plotted. Between-group comparisons were performed using the log-rank test. Follow-up time was defined as the interval (in days) from the baseline hospitalization date to the date of first CAS diagnosis or the last follow-up date.

#### 2.6.4 Cox proportional hazards regression models

To evaluate the association between each composite index and incident CAS, three Cox proportional hazards regression models with stepwise adjustment for confounders were constructed.

##### 2.6.4.1 Model 1 (crude model)

Unadjusted model, including only the composite index, showing the crude association.

##### 2.6.4.2 Model 2

Adjusted for sex.

##### 2.6.4.3 Model 3 (fully adjusted model)

Adjusted for sex, hypertension, CAD, dyslipidemia, DKD, CKD, and NAFLD.

All Cox models used the first quartile (Q1) of each composite index as the reference group, and results were presented as hazard ratios (HRs) with 95% confidence intervals (CIs).

#### 2.6.5 Proportional hazards assumption testing

The proportional hazards (PH) assumption was assessed using Schoenfeld residuals. Preliminary analysis showed that the global PH test for the fully adjusted model was significant (P < 0.001), indicating time-varying effects for some covariates. Therefore, a time-dependent coefficient model was used as the primary analytical approach, incorporating the tt() function with a log(time + 1) transformation to allow HRs to vary over time, without requiring the proportional hazards assumption. This provides more robust effect estimates. As sensitivity analyses, results from stratified Cox models (stratified by CAD, CKD, and NAFLD) and traditional Cox models (Models 1–3) were also reported.

#### 2.6.6 Sensitivity analyses

##### 2.6.6.1 Sensitivity analysis 1 (stratified Cox model)

Stratified by CAD, CKD, and NAFLD, and adjusted for sex, hypertension, dyslipidemia, and DKD, to evaluate the robustness of results within these strata.

##### 2.6.6.2 Sensitivity analysis 2 (traditional Cox model)

Results from traditional Cox regression models (Models 1–3) were reported as **Supplementary Material** for comparison with conventional methods.

#### 2.6.7 Multiple comparison correction

Given the testing of 17 composite indices simultaneously, there was a risk of type I error inflation. The Benjamini–Hochberg false discovery rate (FDR) method was applied for multiple comparison correction, with Q < 0.05 considered statistically significant.

#### 2.6.8 Multicollinearity diagnostics

Variance inflation factor (VIF) was used to assess multicollinearity among covariates, with VIF > 5 indicating moderate collinearity. All models used robust standard errors (robust = TRUE) to enhance result robustness.

#### 2.6.9 Subgroup analyses

To assess the stability of the associations across different populations, subgroup analyses were performed stratified by: (1) age group: <60, 60–74, and ≥75 years; (2) sex group: male and female. Subgroup analyses used the fully adjusted model (Model 3) and reported HRs and 95% CIs for Q1–Q4 within each subgroup. Interaction terms between the exposure variable and subgroup variables were introduced to test for effect modification.

#### 2.6.10 Significance level

All tests were two-sided, with P < 0.05 considered statistically significant; after FDR correction, Q < 0.05 was considered statistically significant.

## 3 Results

### 3.1 Baseline characteristics of the study population

A total of 15,492 patients with T2DM were included in this study. The median follow-up time was 306.5 days (IQR: 92–722 days), with a maximum follow-up of 2,037 days. During follow-up, 1,841 patients (11.88%) developed incident CAS.

Baseline characteristic comparisons (**Table 1**) showed significant differences between patients who developed CAS and those who did not in terms of sex distribution, and the prevalence of hypertension, CAD, dyslipidemia, DKD, CKD, and NAFLD (all P < 0.05). Regarding composite indices, the quartile distributions of BMI, eGFR, albumin, uric acid, SII, SIRI, NLR, PLR, NHR, and MHR differed significantly between the CAS and non-CAS groups (all P < 0.05). However, no significant differences were observed between the two groups in age, AIP, TyG HbA1c, CRI-I, LDL/HDL ratio, non-HDL-C, AI, or LCI (all P > 0.05).

**Table 1:** Baseline characteristics of the study population.

Variables	Total (n=15492)	N-CAS (n=13651)	CAS (n=1841)	Statistic	*P*
BMI,n (%)				χ²=19.86	<0.001
Q1	4492 (29.00)	4035 (29.56)	457 (24.82)		
Q2	3440 (22.21)	3030 (22.20)	410 (22.27)		
Q3	4251 (27.44)	3699 (27.10)	552 (29.98)		
Q4	3309 (21.36)	2887 (21.15)	422 (22.92)		
eGFR Q4, n (%)				χ²=14.31	0.003
Q1	3873 (25.00)	3476 (25.46)	397 (21.56)		
Q2	3873 (25.00)	3407 (24.96)	466 (25.31)		
Q3	3876 (25.02)	3379 (24.75)	497 (27.00)		
Q4	3870 (24.98)	3389 (24.83)	481 (26.13)		
Albumin Q4, n (%)				χ²=127.47	<0.001
Q1	3873 (25.00)	3585 (26.26)	288 (15.64)		
Q2	3879 (25.04)	3450 (25.27)	429 (23.30)		
Q3	3872 (24.99)	3318 (24.31)	554 (30.09)		
Q4	3868 (24.97)	3298 (24.16)	570 (30.96)		
Uric acid Q4, n (%)				χ²=89.72	<0.001
Q1	3873 (25.00)	3557 (26.06)	316 (17.16)		
Q2	3873 (25.00)	3420 (25.05)	453 (24.61)		
Q3	3873 (25.00)	3382 (24.77)	491 (26.67)		
Q4	3873 (25.00)	3292 (24.12)	581 (31.56)		
SII Q4, n (%)				χ²=45.90	<0.001
Q1	3873 (25.00)	3384 (24.79)	489 (26.56)		
Q2	3873 (25.00)	3338 (24.45)	535 (29.06)		
Q3	3873 (25.00)	3406 (24.95)	467 (25.37)		
Q4	3873 (25.00)	3523 (25.81)	350 (19.01)		
SIRI Q4, n (%)				χ²=34.58	<0.001
Q1	3873 (25.00)	3372 (24.70)	501 (27.21)		
Q2	3873 (25.00)	3385 (24.80)	488 (26.51)		
Q3	3873 (25.00)	3379 (24.75)	494 (26.83)		
Q4	3873 (25.00)	3515 (25.75)	358 (19.45)		
NLR Q4, n (%)				χ²=64.77	<0.001
Q1	3873 (25.00)	3346 (24.51)	527 (28.63)		
Q2	3873 (25.00)	3347 (24.52)	526 (28.57)		
Q3	3873 (25.00)	3413 (25.00)	460 (24.99)		
Q4	3873 (25.00)	3545 (25.97)	328 (17.82)		
PLR Q4, n (%)				χ²=65.20	<0.001
Q1	3873 (25.00)	3357 (24.59)	516 (28.03)		
Q2	3875 (25.01)	3353 (24.56)	522 (28.35)		
Q3	3871 (24.99)	3390 (24.83)	481 (26.13)		
Q4	3873 (25.00)	3551 (26.01)	322 (17.49)		
NHR Q4, n (%)				χ²=10.80	0.013
Q1	3873 (25.00)	3437 (25.18)	436 (23.68)		
Q2	3873 (25.00)	3366 (24.66)	507 (27.54)		
Q3	3873 (25.00)	3398 (24.89)	475 (25.80)		
Q4	3873 (25.00)	3450 (25.27)	423 (22.98)		
MHR Q4, n (%)				χ²=8.65	0.034
Q1	3878 (25.03)	3463 (25.37)	415 (22.54)		
Q2	3944 (25.46)	3461 (25.35)	483 (26.24)		
Q3	3801 (24.54)	3315 (24.28)	486 (26.40)		
Q4	3869 (24.97)	3412 (24.99)	457 (24.82)		
AIP index Q4, n (%)				χ²=1.69	0.638
Q1	3873 (25.00)	3426 (25.10)	447 (24.28)		
Q2	3873 (25.00)	3407 (24.96)	466 (25.31)		
Q3	3873 (25.00)	3394 (24.86)	479 (26.02)		
Q4	3873 (25.00)	3424 (25.08)	449 (24.39)		
TyG HbA1c Q4, n (%)				χ²=5.13	0.163
Q1	3873 (25.00)	3416 (25.02)	457 (24.82)		
Q2	3876 (25.02)	3449 (25.27)	427 (23.19)		
Q3	3871 (24.99)	3404 (24.94)	467 (25.37)		
Q4	3872 (24.99)	3382 (24.77)	490 (26.62)		
CRI I Q4, n (%)				χ²=3.16	0.367
Q1	3873 (25.00)	3393 (24.86)	480 (26.07)		
Q2	3874 (25.01)	3434 (25.16)	440 (23.90)		
Q3	3872 (24.99)	3427 (25.10)	445 (24.17)		
Q4	3873 (25.00)	3397 (24.88)	476 (25.86)		
LDL HDL ratio Q4, n (%)				χ²=5.11	0.164
Q1	3873 (25.00)	3380 (24.76)	493 (26.78)		
Q2	3884 (25.07)	3454 (25.30)	430 (23.36)		
Q3	3863 (24.94)	3403 (24.93)	460 (24.99)		
Q4	3872 (24.99)	3414 (25.01)	458 (24.88)		
Non-HDL-C Q4, n (%)				χ²=4.01	0.260
Q1	3895 (25.14)	3431 (25.13)	464 (25.20)		
Q2	3904 (25.20)	3415 (25.02)	489 (26.56)		
Q3	3847 (24.83)	3421 (25.06)	426 (23.14)		
Q4	3846 (24.83)	3384 (24.79)	462 (25.10)		
AI Q4, n (%)				χ²=3.16	0.367
Q1	3873 (25.00)	3393 (24.86)	480 (26.07)		
Q2	3874 (25.01)	3434 (25.16)	440 (23.90)		
Q3	3872 (24.99)	3427 (25.10)	445 (24.17)		
Q4	3873 (25.00)	3397 (24.88)	476 (25.86)		
LCI Q4, n (%)				χ²=0.75	0.861
Q1	3873 (25.00)	3408 (24.97)	465 (25.26)		
Q2	3873 (25.00)	3419 (25.05)	454 (24.66)		
Q3	3873 (25.00)	3401 (24.91)	472 (25.64)		
Q4	3873 (25.00)	3423 (25.08)	450 (24.44)		
Age, M (Q_1_, Q_3_)	63.00 (55.00,71.00)	63.00 (55.00,71.00)	62.00 (56.00,69.00)	Z=-0.62	0.533
Sex, n (%)				χ²=29.34	<0.001
Male	9663 (62.37)	8409 (61.60)	1254 (68.12)		
Female	5829 (37.63)	5242 (38.40)	587 (31.88)		
Marital status, n (%)				χ²=0.59	0.443
Married	14295 (92.27)	12588 (92.21)	1707 (92.72)		
Divorced/Widowed/Unmarried	1197 (7.73)	1063 (7.79)	134 (7.28)		
Hypertension, n (%)				χ²=36.95	<0.001
No	5461 (35.25)	4929 (36.11)	532 (28.90)		
Yes	10031 (64.75)	8722 (63.89)	1309 (71.10)		
CAD, n (%)				χ²=197.26	<0.001
No	9228 (59.57)	8409 (61.60)	819 (44.49)		
Yes	6264 (40.43)	5242 (38.40)	1022 (55.51)		
Dyslipidemia, n (%)				χ²=10.88	<0.001
No	14570 (94.05)	12870 (94.28)	1700 (92.34)		
Yes	922 (5.95)	781 (5.72)	141 (7.66)		
Hyperuricemia, n (%)				χ²=0.88	0.349
No	15230 (98.31)	13425 (98.34)	1805 (98.04)		
Yes	262 (1.69)	226 (1.66)	36 (1.96)		
Diabetes nephropathy, n (%)				χ²=8.26	0.004
No	14735 (95.11)	12959 (94.93)	1776 (96.47)		
Yes	757 (4.89)	692 (5.07)	65 (3.53)		
CKD, n (%)				χ²=17.92	<0.001
No	14312 (92.38)	12566 (92.05)	1746 (94.84)		
Yes	1180 (7.62)	1085 (7.95)	95 (5.16)		
Proteinuria, n (%)				χ²=0.82	0.365
No	15383 (99.30)	13558 (99.32)	1825 (99.13)		
Yes	109 (0.70)	93 (0.68)	16 (0.87)		
NAFLD, n (%)				χ²=40.79	<0.001
No	12478 (80.54)	11097 (81.29)	1381 (75.01)		
Yes	3014 (19.46)	2554 (18.71)	460 (24.99)		

Z: Mann-Whitney test, χ²: Chi-square test.

M: Median, Q₁: 1st Quartile, Q₃: 3rd Quartile.

Bold: P < 0.05.

### 3.2 Cox regression analysis of composite indices and incident carotid atherosclerosis

#### 3.2.1 Proportional hazards assumption testing and modeling strategy

In testing the proportional hazards assumption for the fully adjusted model using Schoenfeld residuals, the global test for the original model was significant (**Supplementary Table 1**: Global P < 0.001), indicating time-varying effects for some covariates. A time-dependent coefficient model was therefore used as the primary analytical approach, which does not require the proportional hazards assumption, providing more robust effect estimates. As sensitivity analyses, we also report results from stratified Cox models (stratified by CAD, CKD, and NAFLD) and traditional Cox models (Models 1–3).

#### 3.2.2 Primary analysis: time-dependent coefficient model

Results from the time-dependent coefficient model (**Table 2**) showed that after full adjustment for confounders (sex, hypertension, CAD, dyslipidemia, DKD, CKD, NAFLD), higher quartile levels were significantly associated with an increased risk of incident CAS for SII (HR per quartile = 1.38, 95% CI: 1.10–1.73, P = 0.0046), SIRI (HR = 1.37, 95% CI: 1.10–1.72, P = 0.0061), NLR (HR = 1.40, 95% CI: 1.12–1.75, P = 0.0028), and NHR (HR = 1.34, 95% CI: 1.07–1.67, P = 0.0115). Albumin was inversely associated with incident CAS (HR per quartile = 0.74, 95% CI: 0.59–0.93, P = 0.0102).

**Table 2:** Time-dependent coefficient model of composite indices and incident carotid atherosclerosis (overall population).

Variable	HR (per quartile)	95% CI	P_raw	P_FDR	P_time_interaction
BMI	1.25	1.25 (1.00-1.56)	0.0528	0.0744	**0.0326**
eGFR	0.80	0.80 (0.64-1.00)	0.0525	0.0744	0.0785
Albumin	0.74	0.74 (0.59-0.93)	**0.0102**	**0.023**	**0.0045**
Uric acid	0.82	0.82 (0.66-1.02)	0.0596	0.0744	0.069
SII	1.38	1.38 (1.10-1.73)	**0.0046**	**0.0202**	**0.0117**
SIRI	1.37	1.37 (1.10-1.72)	**0.0061**	**0.0202**	**0.0145**
NLR	1.40	1.40 (1.12-1.75)	**0.0028**	**0.0202**	**0.0028**
PLR	1.17	1.17 (0.94-1.47)	0.1626	0.1626	0.1376
NHR	1.34	1.34 (1.07-1.67)	**0.0115**	**0.023**	**0.0283**
MHR	1.18	1.18 (0.94-1.48)	0.1511	0.1626	0.2911

Bold: P < 0.05.

These associations remained robust after Benjamini–Hochberg FDR correction (Q < 0.05). Additionally, the time interaction terms were significant for BMI (P = 0.0326), albumin (P = 0.0045), SII (P = 0.0117), SIRI (P = 0.0145), NLR (P = 0.0028), and NHR (P = 0.0283) (**Table 2**), further confirming the necessity of using a time-dependent model. BMI, eGFR, uric acid, PLR, and MHR did not reach statistical significance in the primary analysis (all P > 0.05).

#### 3.2.3 Sensitivity analyses

##### 3.2.3.1 Sensitivity analysis 1 (stratified Cox model)

After stratification by CAD, CKD, and NAFLD (**Table 3**), results were largely consistent with the primary analysis. In the fully adjusted model (adjusted for sex, hypertension, dyslipidemia, and DKD), SII (Q3 vs. Q1: HR = 1.18, 95% CI: 1.04–1.34, P = 0.0121), SIRI (Q3 vs. Q1: HR = 1.15, 95% CI: 1.02–1.31, P = 0.0278), NHR (Q4 vs. Q1: HR = 1.16, 95% CI: 1.01–1.33, P = 0.0355), and MHR (Q4 vs. Q1: HR = 1.17, 95% CI: 1.02–1.34, P = 0.0247) remained significantly associated with an increased risk of incident CAS. Albumin (Q3 vs. Q1: HR = 1.18, 95% CI: 1.02–1.37, P = 0.0253) also showed a positive association. BMI, NLR, PLR, uric acid, and eGFR showed no independent associations in any model.

**Table 3:** Sensitivity analysis 1: stratified Cox model (strata: CAD, CKD, NAFLD) adjusted for sex + hypertension + dyslipidemia + DKD.

Variable	Group	HR_CI	P
Albumin	Q1	1.00 (Reference)	
	Q2	1.05 (0.90-1.22)	0.5533
	Q3	1.18 (1.02-1.37)	**0.0253**
	Q4	1.05 (0.91-1.22)	0.4858
BMI	Q1	1.00 (Reference)	
	Q2	0.90 (0.78-1.02)	0.1075
	Q3	0.93 (0.82-1.06)	0.2818
	Q4	0.94 (0.82-1.07)	0.3626
MHR	Q1	1.00 (Reference)	
	Q2	1.05 (0.92-1.20)	0.4724
	Q3	1.07 (0.94-1.22)	0.3138
	Q4	1.17 (1.02-1.34)	**0.0247**
NHR	Q1	1.00 (Reference)	
	Q2	1.12 (0.98-1.27)	0.0904
	Q3	1.12 (0.98-1.28)	0.0868
	Q4	1.16 (1.01-1.33)	**0.0355**
NLR	Q1	1.00 (Reference)	
	Q2	1.06 (0.94-1.20)	0.3292
	Q3	1.12 (0.98-1.27)	0.0874
	Q4	0.97 (0.84-1.12)	0.6922
PLR	Q1	1.00 (Reference)	
	Q2	1.06 (0.94-1.20)	0.3097
	Q3	1.08 (0.96-1.23)	0.2001
	Q4	0.95 (0.82-1.09)	0.4496
SII	Q1	1.00 (Reference)	
	Q2	1.14 (1.01-1.29)	**0.0314**
	Q3	1.18 (1.04-1.34)	**0.0121**
	Q4	1.11 (0.97-1.28)	0.1287
SIRI	Q1	1.00 (Reference)	
	Q2	0.98 (0.86-1.11)	0.7305
	Q3	1.15 (1.02-1.31)	**0.0278**
	Q4	1.07 (0.93-1.23)	0.3485
Uric acid	Q1	1.00 (Reference)	
	Q2	1.12 (0.96-1.29)	0.1407
	Q3	1.14 (0.99-1.32)	0.0715
	Q4	1.00 (0.86-1.15)	0.9631
eGFR	Q1	1.00 (Reference)	
	Q2	1.03 (0.90-1.18)	0.6793
	Q3	0.96 (0.84-1.10)	0.5692
	Q4	0.92 (0.80-1.07)	0.281

HR, Hazard Ratio; CI, Confidence Interval.

Bold, P < 0.05.

##### 3.2.3.2 Sensitivity analysis 2 (traditional Cox model)

As shown in the **Supplementary Material** (**Supplementary Table 3**), the traditional Cox model (fully adjusted Model 3) showed that SII (Q3 vs. Q1: HR = 1.17, 95% CI: 1.03–1.33, P = 0.0159), SIRI (Q3 vs. Q1: HR = 1.14, 95% CI: 1.01–1.30, P = 0.0388), NHR (Q4 vs. Q1: HR = 1.16, 95% CI: 1.01–1.32, P = 0.0348), MHR (Q4 vs. Q1: HR = 1.16, 95% CI: 1.02–1.33, P = 0.0295), and albumin (Q3 vs. Q1: HR = 1.19, 95% CI: 1.03–1.37, P = 0.0205) were significantly associated with incident CAS, further supporting the robustness of the primary findings. Notably, uric acid showed significant associations in Models 1 and 2, but this association disappeared after full adjustment in Model 3 (Q4 vs. Q1: HR = 1.00, 95% CI: 0.87–1.15, P = 0.9812).

### 3.3 Subgroup analyses: effect modification by age and sex

#### 3.3.1 Age subgroups

Age-stratified analysis (**Table 4**) showed varying patterns of association across age groups.

**Table 4:** Cox regression analysis of composite indices and incident carotid atherosclerosis (age subgroups).

			Model 1		Model 2		Model 3:		FDR (Model 3)
Subgroup	Variable	Group	HR (95% CI)	P value	HR (95% CI)	P value	HR (95% CI)	P value	P_FDR
<60 years	BMI	Q1	1.00 (Reference)		1.00 (Reference)		1.00 (Reference)		
	BMI	Q2	1.04 (0.83-1.31)	0.7341	1.01 (0.8-1.27)	0.9549	0.95 (0.75-1.19)	0.6342	0.7957
	BMI	Q3	1.07 (0.86-1.33)	0.5228	1.04 (0.84-1.29)	0.7333	0.91 (0.73-1.13)	0.4006	0.7957
	BMI	Q4	1.12 (0.9-1.39)	0.3063	1.1 (0.88-1.36)	0.4042	0.92 (0.73-1.16)	0.4776	0.7957
	eGFR	Q1	1.00 (Reference)		1.00 (Reference)		1.00 (Reference)		
	eGFR	Q2	1.09 (0.81-1.47)	0.5804	1.11 (0.82-1.51)	0.4818	1.06 (0.77-1.46)	0.7395	0.8216
	eGFR	Q3	1.1 (0.83-1.45)	0.5114	1.1 (0.83-1.45)	0.5079	1.07 (0.79-1.45)	0.6687	0.7957
	eGFR	Q4	1 (0.77-1.3)	0.9839	1.02 (0.79-1.33)	0.8608	1.01 (0.76-1.34)	0.9601	0.9733
	Albumin	Q1	1.00 (Reference)		1.00 (Reference)		1.00 (Reference)		
	Albumin	Q2	1.23 (0.93-1.61)	0.1492	1.22 (0.93-1.61)	0.1525	1.12 (0.85-1.49)	0.426	0.7957
	Albumin	Q3	1.53 (1.18-1.97)	**0.0012**	1.52 (1.17-1.96)	**0.0014**	1.3 (1-1.7)	0.0514	0.445
	Albumin	Q4	1.28 (1-1.65)	0.0524	1.26 (0.98-1.62)	0.0746	1.07 (0.82-1.4)	0.6021	0.7957
	Uric acid	Q1	1.00 (Reference)		1.00 (Reference)		1.00 (Reference)		
	Uric acid	Q2	1.41 (1.11-1.8)	**0.0055**	1.35 (1.06-1.73)	**0.0151**	1.24 (0.97-1.58)	0.089	0.445
	Uric acid	Q3	1.33 (1.04-1.69)	**0.0235**	1.23 (0.96-1.58)	0.0997	1.07 (0.83-1.38)	0.5975	0.7957
	Uric acid	Q4	1.13 (0.9-1.43)	0.2823	1.06 (0.84-1.34)	0.6099	0.92 (0.72-1.17)	0.5082	0.7957
	SII	Q1	1.00 (Reference)		1.00 (Reference)		1.00 (Reference)		
	SII	Q2	1.1 (0.91-1.34)	0.3269	1.1 (0.91-1.34)	0.3328	1.07 (0.88-1.3)	0.5243	0.7957
	SII	Q3	1.26 (1.03-1.54)	**0.0266**	1.27 (1.03-1.55)	**0.022**	1.24 (1.01-1.53)	**0.0368**	0.445
	SII	Q4	1.01 (0.8-1.27)	0.9432	1.02 (0.81-1.28)	0.8681	0.98 (0.78-1.24)	0.8918	0.9555
	SIRI	Q1	1.00 (Reference)		1.00 (Reference)		1.00 (Reference)		
	SIRI	Q2	1.09 (0.9-1.32)	0.3763	1.05 (0.86-1.27)	0.6398	1.05 (0.86-1.27)	0.6466	0.7957
	SIRI	Q3	1.33 (1.09-1.62)	**0.0053**	1.27 (1.04-1.55)	**0.0168**	1.26 (1.03-1.55)	**0.0234**	0.445
	SIRI	Q4	0.94 (0.74-1.19)	0.59	0.89 (0.7-1.13)	0.3544	0.91 (0.72-1.17)	0.4702	0.7957
	NLR	Q1	1.00 (Reference)		1.00 (Reference)		1.00 (Reference)		
	NLR	Q2	1.11 (0.93-1.34)	0.2564	1.09 (0.91-1.32)	0.3436	1.11 (0.92-1.34)	0.2752	0.7957
	NLR	Q3	1.13 (0.92-1.38)	0.2527	1.1 (0.9-1.35)	0.3631	1.15 (0.94-1.42)	0.1786	0.7654
	NLR	Q4	0.79 (0.62-1)	0.0529	0.77 (0.61-0.98)	**0.0344**	0.79 (0.62-1.01)	0.0595	0.445
	PLR	Q1	1.00 (Reference)		1.00 (Reference)		1.00 (Reference)		
	PLR	Q2	0.94 (0.77-1.15)	0.5568	0.96 (0.79-1.17)	0.6964	0.94 (0.77-1.15)	0.5666	0.7957
	PLR	Q3	1 (0.82-1.21)	0.9802	1.03 (0.84-1.25)	0.7879	1.04 (0.85-1.27)	0.6896	0.7957
	PLR	Q4	0.87 (0.69-1.09)	0.2336	0.91 (0.72-1.14)	0.405	0.94 (0.75-1.19)	0.614	0.7957
	NHR	Q1	1.00 (Reference)		1.00 (Reference)		1.00 (Reference)		
	NHR	Q2	1.22 (0.99-1.51)	0.0601	1.19 (0.97-1.48)	0.1009	1.1 (0.88-1.36)	0.404	0.7957
	NHR	Q3	1.23 (0.99-1.52)	0.059	1.19 (0.96-1.47)	0.1171	1.07 (0.86-1.34)	0.5204	0.7957
	NHR	Q4	1.26 (1.01-1.57)	**0.0415**	1.2 (0.96-1.5)	0.105	1.07 (0.85-1.34)	0.5673	0.7957
	MHR	Q1	1.00 (Reference)		1.00 (Reference)		1.00 (Reference)		
	MHR	Q2	1.03 (0.82-1.28)	0.8202	0.99 (0.79-1.24)	0.9384	0.94 (0.75-1.18)	0.6128	0.7957
	MHR	Q3	1.15 (0.92-1.43)	0.2223	1.09 (0.87-1.35)	0.47	1 (0.8-1.25)	0.9733	0.9733
	MHR	Q4	1.41 (1.13-1.75)	**0.002**	1.31 (1.05-1.64)	**0.0162**	1.22 (0.97-1.53)	0.087	0.445
60–74 years	BMI	Q1	1.00 (Reference)		1.00 (Reference)		1.00 (Reference)		
	BMI	Q2	0.93 (0.77-1.11)	0.4179	0.9 (0.75-1.08)	0.2721	0.85 (0.71-1.03)	0.0938	0.3519
	BMI	Q3	1.03 (0.87-1.22)	0.6983	1 (0.84-1.18)	0.9987	0.92 (0.78-1.1)	0.362	0.5545
	BMI	Q4	1.02 (0.84-1.23)	0.8461	1.01 (0.84-1.22)	0.9039	0.92 (0.76-1.11)	0.3759	0.5545
	eGFR	Q1	1.00 (Reference)		1.00 (Reference)		1.00 (Reference)		
	eGFR	Q2	1.09 (0.91-1.3)	0.353	1.09 (0.92-1.3)	0.3287	1.03 (0.86-1.23)	0.772	0.8272
	eGFR	Q3	0.91 (0.77-1.09)	0.3161	0.92 (0.77-1.1)	0.3482	0.88 (0.74-1.06)	0.18	0.4058
	eGFR	Q4	0.98 (0.79-1.21)	0.8475	0.97 (0.78-1.2)	0.7629	0.95 (0.76-1.19)	0.6615	0.7615
	Albumin	Q1	1.00 (Reference)		1.00 (Reference)		1.00 (Reference)		
	Albumin	Q2	0.98 (0.79-1.2)	0.8129	0.97 (0.79-1.19)	0.756	0.91 (0.74-1.12)	0.3605	0.5545
	Albumin	Q3	1.19 (0.98-1.45)	0.0825	1.19 (0.98-1.44)	0.0864	1.09 (0.89-1.33)	0.39	0.5545
	Albumin	Q4	1.12 (0.92-1.35)	0.2723	1.1 (0.91-1.34)	0.3337	1.01 (0.83-1.23)	0.9119	0.9119
	Uric acid	Q1	1.00 (Reference)		1.00 (Reference)		1.00 (Reference)		
	Uric acid	Q2	1.17 (0.96-1.43)	0.1246	1.14 (0.93-1.39)	0.1995	1.09 (0.89-1.33)	0.4185	0.5545
	Uric acid	Q3	1.35 (1.11-1.64)	**0.0025**	1.28 (1.05-1.56)	**0.013**	1.23 (1.01-1.49)	**0.0421**	0.3519
	Uric acid	Q4	1.2 (1-1.45)	0.0529	1.15 (0.95-1.4)	0.138	1.06 (0.87-1.28)	0.59	0.708
	SII	Q1	1.00 (Reference)		1.00 (Reference)		1.00 (Reference)		
	SII	Q2	1.23 (1.04-1.46)	**0.0173**	1.22 (1.03-1.45)	**0.0208**	1.16 (0.98-1.37)	0.0921	0.3519
	SII	Q3	1.18 (0.98-1.41)	0.0753	1.17 (0.98-1.4)	0.0814	1.13 (0.94-1.35)	0.2025	0.4058
	SII	Q4	1.17 (0.96-1.41)	0.1132	1.16 (0.96-1.4)	0.1255	1.17 (0.97-1.42)	0.1062	0.354
	SIRI	Q1	1.00 (Reference)		1.00 (Reference)		1.00 (Reference)		
	SIRI	Q2	1.04 (0.87-1.24)	0.6347	1 (0.84-1.2)	0.9571	0.96 (0.8-1.15)	0.6853	0.7615
	SIRI	Q3	1.21 (1.02-1.45)	**0.0316**	1.17 (0.98-1.4)	0.0749	1.14 (0.96-1.37)	0.1429	0.4058
	SIRI	Q4	1.28 (1.07-1.55)	**0.0079**	1.22 (1.01-1.47)	**0.0383**	1.25 (1.03-1.52)	**0.021**	0.3519
	NLR	Q1	1.00 (Reference)		1.00 (Reference)		1.00 (Reference)		
	NLR	Q2	1.06 (0.89-1.26)	0.5126	1.03 (0.87-1.23)	0.7186	1.02 (0.86-1.21)	0.8371	0.866
	NLR	Q3	1.11 (0.93-1.32)	0.2342	1.08 (0.9-1.28)	0.4106	1.08 (0.9-1.29)	0.4251	0.5545
	NLR	Q4	1.09 (0.91-1.32)	0.3474	1.06 (0.88-1.29)	0.5213	1.12 (0.92-1.36)	0.2488	0.4665
	PLR	Q1	1.00 (Reference)		1.00 (Reference)		1.00 (Reference)		
	PLR	Q2	1.15 (0.97-1.36)	0.1144	1.15 (0.97-1.37)	0.0979	1.12 (0.95-1.33)	0.1816	0.4058
	PLR	Q3	1.11 (0.93-1.33)	0.2329	1.13 (0.95-1.34)	0.1833	1.12 (0.94-1.34)	0.2029	0.4058
	PLR	Q4	0.9 (0.74-1.1)	0.3	0.91 (0.74-1.1)	0.3265	0.94 (0.77-1.15)	0.5489	0.6861
	NHR	Q1	1.00 (Reference)		1.00 (Reference)		1.00 (Reference)		
	NHR	Q2	1.14 (0.96-1.36)	0.1325	1.12 (0.94-1.33)	0.2129	1.08 (0.9-1.29)	0.3937	0.5545
	NHR	Q3	1.21 (1.01-1.44)	**0.0387**	1.17 (0.98-1.4)	0.0792	1.13 (0.94-1.35)	0.1867	0.4058
	NHR	Q4	1.27 (1.06-1.53)	**0.0111**	1.23 (1.02-1.48)	**0.0293**	1.2 (0.99-1.44)	0.0626	0.3519
	MHR	Q1	1.00 (Reference)		1.00 (Reference)		1.00 (Reference)		
	MHR	Q2	1.26 (1.05-1.5)	**0.0119**	1.23 (1.02-1.47)	**0.0267**	1.2 (1.01-1.44)	**0.0436**	0.3519
	MHR	Q3	1.29 (1.07-1.54)	**0.0065**	1.23 (1.03-1.48)	**0.024**	1.18 (0.98-1.42)	0.089	0.3519
	MHR	Q4	1.32 (1.1-1.58)	**0.0034**	1.24 (1.03-1.5)	**0.0239**	1.21 (1-1.47)	**0.047**	0.3519
≥75 years	BMI	Q1	1.00 (Reference)		1.00 (Reference)		1.00 (Reference)		
	BMI	Q2	0.87 (0.59-1.28)	0.4774	0.86 (0.59-1.27)	0.4554	0.86 (0.59-1.27)	0.4502	0.888
	BMI	Q3	1.04 (0.73-1.48)	0.8423	1.03 (0.72-1.46)	0.8898	0.98 (0.68-1.41)	0.9275	0.9275
	BMI	Q4	1.03 (0.69-1.55)	0.878	1.04 (0.69-1.57)	0.8466	0.97 (0.64-1.49)	0.9035	0.9275
	eGFR	Q1	1.00 (Reference)		1.00 (Reference)		1.00 (Reference)		
	eGFR	Q2	0.91 (0.67-1.23)	0.5534	0.92 (0.68-1.24)	0.571	0.94 (0.69-1.28)	0.6808	0.888
	eGFR	Q3	0.86 (0.54-1.37)	0.521	0.86 (0.54-1.38)	0.5369	0.88 (0.54-1.42)	0.5922	0.888
	eGFR	Q4	0.91 (0.4-2.04)	0.817	0.91 (0.41-2.02)	0.8079	0.94 (0.42-2.12)	0.8786	0.9275
	Albumin	Q1	1.00 (Reference)		1.00 (Reference)		1.00 (Reference)		
	Albumin	Q2	1.51 (1.03-2.23)	**0.0369**	1.53 (1.03-2.26)	**0.0331**	1.48 (0.99-2.2)	0.0536	0.8034
	Albumin	Q3	1.23 (0.81-1.86)	0.3281	1.24 (0.82-1.88)	0.3083	1.16 (0.77-1.77)	0.4749	0.888
	Albumin	Q4	1.42 (0.93-2.19)	0.1058	1.44 (0.93-2.21)	0.0984	1.36 (0.88-2.09)	0.1672	0.888
	Uric acid	Q1	1.00 (Reference)		1.00 (Reference)		1.00 (Reference)		
	Uric acid	Q2	1 (0.65-1.56)	0.9827	0.99 (0.64-1.55)	0.9786	0.96 (0.61-1.5)	0.8463	0.9275
	Uric acid	Q3	1.16 (0.76-1.77)	0.4811	1.15 (0.76-1.75)	0.513	1.1 (0.72-1.68)	0.65	0.888
	Uric acid	Q4	1.29 (0.86-1.94)	0.2243	1.27 (0.84-1.92)	0.2585	1.21 (0.78-1.86)	0.3965	0.888
	SII	Q1	1.00 (Reference)		1.00 (Reference)		1.00 (Reference)		
	SII	Q2	1.23 (0.84-1.81)	0.2923	1.23 (0.84-1.81)	0.291	1.17 (0.79-1.72)	0.4386	0.888
	SII	Q3	1.15 (0.78-1.69)	0.4885	1.14 (0.78-1.68)	0.4997	1.15 (0.78-1.69)	0.4707	0.888
	SII	Q4	1.23 (0.82-1.84)	0.3169	1.22 (0.82-1.83)	0.3314	1.21 (0.81-1.82)	0.3503	0.888
	SIRI	Q1	1.00 (Reference)		1.00 (Reference)		1.00 (Reference)		
	SIRI	Q2	0.8 (0.54-1.2)	0.2836	0.78 (0.53-1.17)	0.2343	0.77 (0.52-1.15)	0.2014	0.888
	SIRI	Q3	0.83 (0.57-1.21)	0.3419	0.81 (0.56-1.19)	0.2829	0.82 (0.56-1.2)	0.3096	0.888
	SIRI	Q4	0.77 (0.52-1.16)	0.2153	0.74 (0.49-1.12)	0.1583	0.74 (0.49-1.12)	0.1596	0.888
	NLR	Q1	1.00 (Reference)		1.00 (Reference)		1.00 (Reference)		
	NLR	Q2	1.07 (0.72-1.58)	0.7382	1.06 (0.71-1.57)	0.7792	1.06 (0.71-1.57)	0.7894	0.9275
	NLR	Q3	1.09 (0.73-1.64)	0.6604	1.08 (0.72-1.61)	0.714	1.1 (0.73-1.64)	0.6552	0.888
	NLR	Q4	0.9 (0.59-1.38)	0.6334	0.88 (0.57-1.34)	0.5457	0.9 (0.59-1.38)	0.6219	0.888
	PLR	Q1	1.00 (Reference)		1.00 (Reference)		1.00 (Reference)		
	PLR	Q2	1.13 (0.77-1.65)	0.5327	1.12 (0.76-1.64)	0.5599	1.16 (0.79-1.71)	0.4427	0.888
	PLR	Q3	1.06 (0.73-1.56)	0.7532	1.06 (0.73-1.56)	0.7471	1.1 (0.75-1.62)	0.6211	0.888
	PLR	Q4	0.94 (0.62-1.42)	0.7633	0.93 (0.62-1.41)	0.743	0.98 (0.64-1.49)	0.9104	0.9275
	NHR	Q1	1.00 (Reference)		1.00 (Reference)		1.00 (Reference)		
	NHR	Q2	1.45 (0.99-2.14)	0.058	1.45 (0.99-2.14)	0.0591	1.51 (1.03-2.23)	**0.0362**	0.8034
	NHR	Q3	1.26 (0.85-1.87)	0.2539	1.25 (0.84-1.86)	0.2671	1.26 (0.85-1.87)	0.2522	0.888
	NHR	Q4	1.27 (0.82-1.96)	0.2821	1.24 (0.8-1.93)	0.3276	1.25 (0.81-1.95)	0.3109	0.888
	MHR	Q1	1.00 (Reference)		1.00 (Reference)		1.00 (Reference)		
	MHR	Q2	0.91 (0.62-1.33)	0.6256	0.89 (0.6-1.32)	0.5646	0.89 (0.6-1.32)	0.5666	0.888
	MHR	Q3	0.96 (0.66-1.4)	0.8226	0.95 (0.65-1.38)	0.7735	0.94 (0.65-1.38)	0.7669	0.9275
	MHR	Q4	0.87 (0.59-1.3)	0.5031	0.84 (0.55-1.27)	0.401	0.82 (0.54-1.25)	0.3577	0.888

HR, Hazard Ratio; CI, Confidence Interval.

Model 1: Crude (unadjusted).

Model 2: Adjusted for Sex,.

Model 3: Fully adjusted (Sex + Hypertension + CAD + Dyslipidemia + DKD + CKD + NAFLD).

Bold: P < 0.05.

##### 3.3.1.1 < 60 years group

SIRI showed the most prominent association, with Q3 vs. Q1 HR = 1.26 (95% CI: 1.03–1.55, P = 0.0234), and SII (Q3 vs. Q1: HR = 1.24, 95% CI: 1.01–1.53, P = 0.0368). After FDR correction, Q-values for all significant associations were > 0.05, not reaching the corrected significance threshold.

##### 3.3.1.2 60–74 years group

SIRI (Q4 vs. Q1: HR = 1.25, 95% CI: 1.03–1.52, P = 0.021), MHR (Q4 vs. Q1: HR = 1.21, 95% CI: 1.00–1.47, P = 0.047), and uric acid (Q3 vs. Q1: HR = 1.23, 95% CI: 1.01–1.49, P = 0.0421) were significantly associated with CAS risk (raw P < 0.05). However, none remained significant after FDR correction (all Q > 0.05).

##### 3.3.1.3 ≥ 75 years group

SII and NHR showed more prominent effects. NHR (Q2 vs. Q1: HR = 1.51, 95% CI: 1.03–2.23, P = 0.0362) was significant, but significance was lost after FDR correction (Q = 0.8034).

In all age subgroups, raw P < 0.05 associations did not survive FDR correction (all Q > 0.05), suggesting that these subgroup findings should be considered exploratory. Tests for interaction between age and each composite index were not statistically significant (all P > 0.05).

#### 3.3.2 Sex subgroups

Sex-stratified analysis (**Table 5**) revealed distinct association patterns.

**Table 5:** Cox regression analysis of composite indices and incident carotid atherosclerosis (sex subgroups).

			Model 1		Model 2		FDR (Model 2)
Subgroup	Variable	Group	HR (95% CI)	P value	HR (95% CI)	P value	P_FDR
Male	BMI	Q1	1.00 (Reference)		1.00 (Reference)		
	BMI	Q2	0.94 (0.8-1.11)	0.4555	0.9 (0.76-1.06)	0.1958	0.6991
	BMI	Q3	1.07 (0.92-1.25)	0.3813	0.98 (0.84-1.14)	0.7736	0.8782
	BMI	Q4	1.11 (0.94-1.31)	0.2028	0.99 (0.83-1.17)	0.8959	0.9114
	eGFR	Q1	1.00 (Reference)		1.00 (Reference)		
	eGFR	Q2	1.14 (0.96-1.35)	0.1352	1.08 (0.9-1.28)	0.4093	0.7223
	eGFR	Q3	1.05 (0.89-1.24)	0.5476	1.01 (0.85-1.21)	0.8778	0.9114
	eGFR	Q4	1 (0.85-1.17)	0.9842	0.97 (0.82-1.16)	0.7734	0.8782
	Albumin	Q1	1.00 (Reference)		1.00 (Reference)		
	Albumin	Q2	1.18 (0.98-1.43)	0.0878	1.09 (0.9-1.32)	0.3762	0.7223
	Albumin	Q3	1.37 (1.14-1.64)	**<0.001**	1.22 (1.01-1.47)	**0.0354**	0.4468
	Albumin	Q4	1.29 (1.09-1.54)	**0.004**	1.15 (0.96-1.37)	0.1429	0.6991
	Uric acid	Q1	1.00 (Reference)		1.00 (Reference)		
	Uric acid	Q2	1.15 (0.95-1.38)	0.1426	1.06 (0.88-1.28)	0.5111	0.7666
	Uric acid	Q3	1.16 (0.97-1.39)	0.0947	1.07 (0.89-1.28)	0.4528	0.7547
	Uric acid	Q4	0.99 (0.83-1.18)	0.8863	0.88 (0.74-1.06)	0.1798	0.6991
	SII	Q1	1.00 (Reference)		1.00 (Reference)		
	SII	Q2	1.14 (0.98-1.32)	0.0809	1.1 (0.95-1.27)	0.2094	0.6991
	SII	Q3	1.19 (1.02-1.39)	**0.0266**	1.17 (1-1.37)	**0.0447**	0.4468
	SII	Q4	1.08 (0.92-1.28)	0.3477	1.09 (0.92-1.29)	0.295	0.6991
	SIRI	Q1	1.00 (Reference)		1.00 (Reference)		
	SIRI	Q2	0.98 (0.84-1.14)	0.7548	0.97 (0.83-1.13)	0.665	0.8782
	SIRI	Q3	1.19 (1.02-1.39)	**0.0268**	1.19 (1.02-1.39)	**0.031**	0.4468
	SIRI	Q4	1.01 (0.85-1.19)	0.9126	1.04 (0.88-1.24)	0.6154	0.8782
	NLR	Q1	1.00 (Reference)		1.00 (Reference)		
	NLR	Q2	1.07 (0.92-1.23)	0.3915	1.07 (0.92-1.24)	0.393	0.7223
	NLR	Q3	1.04 (0.9-1.22)	0.5738	1.09 (0.93-1.27)	0.3029	0.6991
	NLR	Q4	0.94 (0.8-1.11)	0.4605	0.99 (0.83-1.18)	0.9114	0.9114
	PLR	Q1	1.00 (Reference)		1.00 (Reference)		
	PLR	Q2	1.04 (0.9-1.2)	0.6412	1.02 (0.88-1.18)	0.7904	0.8782
	PLR	Q3	1.06 (0.92-1.24)	0.4129	1.09 (0.93-1.26)	0.2863	0.6991
	PLR	Q4	0.91 (0.77-1.08)	0.2937	0.97 (0.81-1.15)	0.6904	0.8782
	NHR	Q1	1.00 (Reference)		1.00 (Reference)		
	NHR	Q2	1.13 (0.96-1.32)	0.1465	1.08 (0.92-1.27)	0.3352	0.7183
	NHR	Q3	1.19 (1.02-1.4)	**0.0304**	1.14 (0.97-1.34)	0.1149	0.6991
	NHR	Q4	1.15 (0.98-1.36)	0.091	1.1 (0.94-1.3)	0.2416	0.6991
	MHR	Q1	1.00 (Reference)		1.00 (Reference)		
	MHR	Q2	0.99 (0.83-1.17)	0.88	0.97 (0.82-1.15)	0.7258	0.8782
	MHR	Q3	1.11 (0.94-1.31)	0.227	1.06 (0.9-1.26)	0.4804	0.7586
	MHR	Q4	1.13 (0.96-1.34)	0.1376	1.09 (0.93-1.29)	0.2878	0.6991
Female	BMI	Q1	1.00 (Reference)		1.00 (Reference)		
	BMI	Q2	0.97 (0.78-1.21)	0.7887	0.92 (0.73-1.15)	0.4704	0.7011
	BMI	Q3	0.92 (0.74-1.14)	0.4408	0.86 (0.69-1.08)	0.1899	0.4377
	BMI	Q4	0.89 (0.71-1.12)	0.3167	0.84 (0.66-1.05)	0.1254	0.4341
	eGFR	Q1	1.00 (Reference)		1.00 (Reference)		
	eGFR	Q2	0.94 (0.75-1.17)	0.5735	0.97 (0.77-1.22)	0.7745	0.8936
	eGFR	Q3	0.83 (0.66-1.04)	0.1006	0.9 (0.71-1.14)	0.3866	0.6444
	eGFR	Q4	0.75 (0.59-0.95)	**0.0182**	0.89 (0.68-1.15)	0.3536	0.624
	Albumin	Q1	1.00 (Reference)		1.00 (Reference)		
	Albumin	Q2	1.03 (0.8-1.32)	0.818	0.98 (0.76-1.27)	0.8844	0.9149
	Albumin	Q3	1.2 (0.95-1.52)	0.1316	1.14 (0.9-1.46)	0.2837	0.5319
	Albumin	Q4	0.94 (0.73-1.21)	0.6299	0.9 (0.7-1.16)	0.4264	0.6732
	Uric acid	Q1	1.00 (Reference)		1.00 (Reference)		
	Uric acid	Q2	1.24 (0.98-1.57)	0.0684	1.18 (0.93-1.5)	0.1674	0.4341
	Uric acid	Q3	1.34 (1.05-1.72)	**0.0194**	1.23 (0.96-1.58)	0.1049	0.4341
	Uric acid	Q4	1.39 (1.11-1.73)	**0.0041**	1.21 (0.96-1.53)	0.1043	0.4341
	SII	Q1	1.00 (Reference)		1.00 (Reference)		
	SII	Q2	1.28 (1.03-1.59)	**0.0232**	1.23 (0.99-1.53)	0.0585	0.4341
	SII	Q3	1.24 (0.99-1.56)	0.0588	1.18 (0.93-1.48)	0.1666	0.4341
	SII	Q4	1.19 (0.94-1.53)	0.1548	1.17 (0.92-1.49)	0.2043	0.4377
	SIRI	Q1	1.00 (Reference)		1.00 (Reference)		
	SIRI	Q2	1.06 (0.86-1.31)	0.5677	1.01 (0.81-1.25)	0.9635	0.9635
	SIRI	Q3	1.12 (0.9-1.38)	0.3205	1.03 (0.82-1.28)	0.8178	0.9056
	SIRI	Q4	1.19 (0.93-1.51)	0.1677	1.19 (0.93-1.52)	0.1736	0.4341
	NLR	Q1	1.00 (Reference)		1.00 (Reference)		
	NLR	Q2	1.07 (0.87-1.32)	0.5417	1.04 (0.84-1.28)	0.7483	0.8936
	NLR	Q3	1.24 (1-1.54)	**0.0495**	1.17 (0.94-1.46)	0.1599	0.4341
	NLR	Q4	0.96 (0.74-1.23)	0.7244	0.94 (0.74-1.21)	0.6532	0.8165
	PLR	Q1	1.00 (Reference)		1.00 (Reference)		
	PLR	Q2	1.18 (0.95-1.48)	0.1327	1.15 (0.92-1.43)	0.222	0.444
	PLR	Q3	1.12 (0.9-1.4)	0.3125	1.07 (0.85-1.34)	0.5627	0.7572
	PLR	Q4	0.91 (0.71-1.18)	0.478	0.91 (0.71-1.18)	0.4908	0.7011
	NHR	Q1	1.00 (Reference)		1.00 (Reference)		
	NHR	Q2	1.26 (1.02-1.56)	**0.0302**	1.2 (0.97-1.48)	0.0991	0.4341
	NHR	Q3	1.12 (0.89-1.41)	0.336	1.07 (0.85-1.34)	0.5805	0.7572
	NHR	Q4	1.37 (1.08-1.74)	**0.0084**	1.3 (1.03-1.66)	**0.0302**	0.4341
	MHR	Q1	1.00 (Reference)		1.00 (Reference)		
	MHR	Q2	1.24 (1.01-1.53)	**0.0384**	1.2 (0.98-1.48)	0.0808	0.4341
	MHR	Q3	1.12 (0.9-1.4)	0.3192	1.02 (0.81-1.29)	0.8452	0.9056
	MHR	Q4	1.38 (1.08-1.76)	**0.0094**	1.34 (1.05-1.71)	**0.02**	0.4341

HR, Hazard Ratio; CI, Confidence Interval.

Model 1: Crude (unadjusted).

Model 2: Adjusted for Hypertension + CAD + Dyslipidemia + DKD + CKD + NAFLD.

Bold: P < 0.05.

##### 3.3.2.1 Male patients

SII (Q3 vs. Q1: HR = 1.17, 95% CI: 1.00–1.37, P = 0.0447, Q = 0.4468) and SIRI (Q3 vs. Q1: HR = 1.19, 95% CI: 1.02–1.39, P = 0.031, Q = 0.4468) were significantly associated with incident CAS risk (raw P < 0.05). Albumin (Q3 vs. Q1: HR = 1.22, 95% CI: 1.01–1.47, P = 0.0354, Q = 0.4468) also showed a significant positive association.

##### 3.3.2.2 Female patients

NHR (Q4 vs. Q1: HR = 1.30, 95% CI: 1.03–1.66, P = 0.0302, Q = 0.4341) and MHR (Q4 vs. Q1: HR = 1.34, 95% CI: 1.05–1.71, P = 0.02, Q = 0.4341) were significantly associated with an increased risk of incident CAS.

After FDR correction, all significant associations in sex subgroups had Q-values > 0.05, not reaching the corrected significance threshold. Tests for interaction between sex and each composite index were not statistically significant (all P > 0.05).

### 3.4 Survival curve analysis

**Figure 2:**
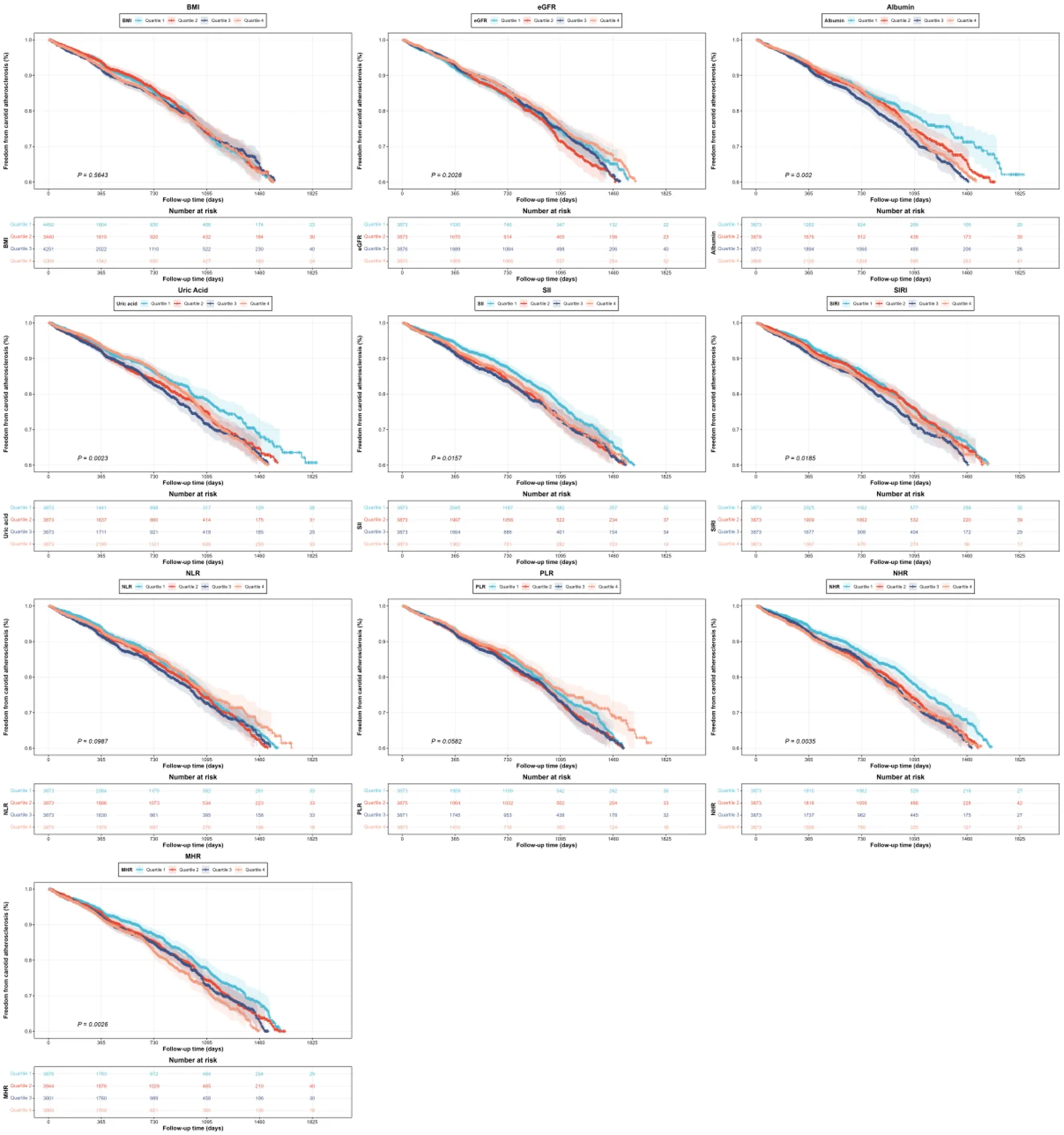
Kaplan–Meier survival curves for cumulative incidence of carotid atherosclerosis according to quartiles of composite indices.

Kaplan–Meier survival analysis (**Figure 2**) further supported the above findings. The log-rank test showed that the cumulative survival without incident CAS differed significantly across quartile groups for albumin (P = 0.002), uric acid (P = 0.002), SII (P = 0.016), SIRI (P = 0.019), NHR (P = 0.004), and MHR (P = 0.003). No significant differences were observed for BMI, eGFR, NLR, or PLR (all P > 0.05).

## 4 Discussion

In this retrospective cohort study of 15,492 T2DM patients, we systematically compared the associations of 17 composite indices with incident CAS. Our findings demonstrated that, after comprehensive adjustment for multiple confounders, SII, SIRI, NLR, and NHR showed significant positive associations, while albumin showed a significant inverse association, while indices solely reflecting glucose metabolism, renal function, or traditional lipid metabolism did not reach statistical significance in fully adjusted models. This aligns with Ross’s “inflammatory theory of atherosclerosis” (13), suggesting that inflammation remains a dominant pathway in CAS development, beyond the effects of hyperglycemia or insulin resistance per se (14). This also implies that assessment of systemic inflammatory status may be more critical for CAS risk stratification in T2DM than traditional metabolic indicators. The inflammatory theory of atherosclerosis has evolved from pathological consensus to a target for clinical intervention (13, 15).

Our analysis found that SII and SIRI were independent correlates of CAS in T2DM patients, consistent with findings from You et al. (16) and Nai et al. (17). As multi-cell integrated inflammatory indices, the significant associations of SII and SIRI reflect the synergistic roles of innate and adaptive immunity in the initiation and progression of atherosclerosis (18). Neutrophils can directly damage vascular endothelium through the release of neutrophil extracellular traps and reactive oxygen species (19); monocytes migrating into the subendothelium and differentiating into foam cells are central to plaque formation (20); and lymphocytes play a bidirectional role in modulating inflammatory responses (21). The independent associations of these indices in T2DM patients suggest that the imbalance among multiple inflammatory cell subsets may more precisely reflect the pathological burden of atherosclerosis than abnormalities in any single cell count.

We also observed prominent associations for the inflammation-lipid ratio indices NHR and MHR. Wang et al. (22), in a multicenter study based on the Chinese Jinshan cohort and the UK Biobank, confirmed that NHR and MHR were independently associated with atherosclerotic event risk in T2DM patients (NHR: OR = 1.24, 95% CI: 1.11–1.39; MHR: OR = 1.22, 95% CI: 1.08–1.37). Song et al. (12) also reported that NHR and MHR were independent risk factors for vascular complications in T2DM. Boughanem et al. (23) demonstrated that NHR was significantly and independently associated with carotid IMT (OR = 1.96, 95% CI: 1.09–3.55). These inflammation-lipid ratio indices combine pro-inflammatory cell counts with the anti-inflammatory lipoprotein HDL-C (24). HDL-C is not only involved in reverse cholesterol transport but also possesses multiple protective functions, including anti-inflammatory, antioxidant, and anti-platelet aggregation effects (25). Elevated NHR and MHR may not simply reflect enhanced pro-inflammatory activity, but rather a bidirectional imbalance of increased pro-inflammatory capacity and decreased anti-inflammatory defense (26, 27). This dual “attack-defense” evaluation framework may more closely reflect the complex pathophysiology of CAS in T2DM than traditional single-dimension indicators.

In our study, BMI, uric acid, and TyG indices showed no statistically significant associations after full adjustment. BMI, as a crude indicator of obesity, cannot distinguish between the heterogeneity of fat distribution and function (28, 29). In T2DM patients, the atherogenic effects of visceral adipose tissue and perivascular fat are far greater than those of subcutaneous fat, and the existence of the “obesity paradox” may further dilute the direct association between BMI and CAS. Our results are consistent with a large cross-sectional study by Li et al. (30) involving 1,947 hospitalized T2DM patients, which showed no significant association between serum uric acid levels and the presence of CAS after adjusting for multiple confounders. The TyG index, a commonly used surrogate marker of insulin resistance, may have its association weakened because its components may be partially explained through other mediating pathways (inflammation, endothelial function, etc.) (31). Previous studies on the association between the TyG index and carotid atherosclerosis in T2DM have been inconsistent. Lin et al. (32) found that the association between TyG and CIMT was significantly modified by ACR and age, but whether this effect is stable in other populations remains to be verified. In our study, the TyG index showed no independent association with CAS after full adjustment for other metabolic factors, suggesting limited independent predictive value of the TyG index for CAS in hospitalized T2DM patients.

Traditional lipid ratio indices did not show independent associations. Although T2DM patients often present with typical hypertriglyceridemia and low HDL-C, combination indices based solely on traditional lipid panel components may not capture the qualitative changes in lipoprotein particles associated with T2DM-related lipid disorders. Luciani et al. (33) noted that T2DM exhibits a unique plasma lipoprotein phenotype, characterized not only by changes in lipoprotein quantity but also by qualitative changes in lipoprotein structure, function, and distribution, including post-secretory glycation and oxidative modification of apolipoprotein B particles. Guardiola et al. (34) reported that even when LDL-C is normal or within therapeutic targets, cholesterol still accumulates in the arteries of T2DM patients. Amor et al. (35) demonstrated in newly diagnosed T2DM patients that advanced lipoprotein profiling by NMR can identify atherogenic lipoprotein abnormalities not captured by conventional lipid profiles, and these abnormalities are significantly associated with carotid atherosclerosis.

Age-stratified analysis showed that in younger T2DM patients (<60 years), SIRI exhibited the strongest association, consistent with Nai et al. (17), who found that SIRI had a greater predictive value in populations with lower metabolic burden. SIRI integrates neutrophils, monocytes, and lymphocytes, all of which play key roles in the initiation phase of atherosclerosis. In younger T2DM patients with relatively shorter disease duration and lower cumulative metabolic burden, early inflammatory responses involving multiple cell types may be the primary mechanism driving vascular wall injury. SIRI, by capturing both innate and adaptive immune information, may more sensitively reflect the immune network dysregulation at this stage (36). Conversely, in older patients, SII and NHR showed stronger association trends, consistent with the findings of Cui et al. (37). Older T2DM patients often have a higher baseline level of systemic inflammation, i.e., a state of “inflammaging” (38). Platelet–inflammation interactions and neutrophil-mediated damage may be significantly amplified (39). Platelet activation is not only involved in thrombosis but can also directly modulate inflammatory responses, forming a positive feedback loop with neutrophils that accelerates vascular injury.

Our sex-stratified analysis revealed that SII and SIRI showed stronger associations in male than in female patients, consistent with a prospective cohort study by Tao et al. (36) involving 12,289 Chinese individuals, which reported a significant sex interaction for SIRI with incident CAS, with a more pronounced effect in males. In contrast, NHR and MHR showed stronger associations in female patients. Zhang et al. (40), in a cross-sectional study of 2,109 middle-aged and older Chinese adults, found that the association between MHR and carotid plaque prevalence was particularly significant in females (OR = 5.921, 95% CI: 1.823–19.231, P = 0.003). Li et al. (41), in a study on sex differences in T2DM incidence, confirmed that inflammatory indices such as LHR, MHR, and NHR had significantly stronger associations with T2DM risk in women than in men. The predictive value of inflammation-lipid ratio indices may be more prominent in females, possibly related to sex differences in HDL-C levels, baseline inflammation, and hormonal environments. These sex differences may arise from interactions between biological factors and environmental factors. Biologically, estrogen exerts pleiotropic protective effects on the cardiovascular system, including improving endothelial function, inhibiting inflammatory responses, and regulating lipid metabolism (42).

### 4.1 Limitations

This study has several limitations. First, The single-center retrospective design may have introduced selection bias. Moreover, CAS identification relied on carotid ultrasound examinations performed in clinical practice rather than systematic screening. Patients with more frequent hospitalizations or more pronounced clinical symptoms had a greater chance of undergoing ultrasound examinations, introducing surveillance bias. Second, Although we used multiple imputation for missing data and controlled for a wide range of confounders using three sequential models, residual confounding inherent to retrospective studies may still have affected the robustness of the results. Due to data extraction constraints, we were unable to obtain information on smoking history, exact T2DM duration, baseline blood pressure, specific glucose-lowering medications (especially SGLT2 inhibitors and GLP-1 receptor agonists) and statin regimens and adherence, or acute infections/inflammatory disease status at admission. Third, All composite indices were based on a single baseline measurement, failing to capture their dynamic changes and temporal relationships with incident CAS. Metabolic and inflammatory status in T2DM fluctuates over time; cumulative exposure or longitudinal trajectories may have stronger predictive value than a single measurement. Fourth, Testing 17 composite indices simultaneously carries a risk of type I error inflation. Although we applied FDR correction and framed all results as exploratory. Fifth, The median follow-up of 306 days is relatively short to fully capture the long-term risk of CAS. The generalizability of our conclusions needs to be validated in multicenter, prospective cohort studies with diverse populations.

## 5 Conclusions

In this study, SII, SIRI, NLR, and NHR were independently associated with increased incident CAS risk in T2DM patients, while albumin showed an inverse association. These inflammation-lipid composite indices may facilitate early risk stratification for CAS beyond routine laboratory tests. Age- and sex-stratified analyses suggested potential heterogeneity, though interaction tests were not significant, and subgroup findings remain exploratory.

Given the single-center retrospective design with inherent surveillance bias, unmeasured confounding, and multiple testing, these findings should not be interpreted as causal or as a basis for clinical screening. The clinical utility and optimal thresholds of these indices require validation in standardized, multicenter prospective cohorts.

## Data Availability

The original contributions presented in the study are included in the article/**Supplementary Material**. Further inquiries can be directed to the corresponding author.

## Supplementary material

Supplementary Table 1 Proportional hazards assumption test for the original model (Schoenfeld residuals method).

Supplementary Table 2 Proportional hazards assumption test for the stratified model (sensitivity analysis).

Supplementary Table 3 Sensitivity analysis 2: traditional Cox regression models (Models 1/2/3).
