# Supplementary material for: Type 2 diabetes mellitus and incident carotid atherosclerosis: a retrospective cohort study on composite indices
Source: Front Endocrinol (Lausanne). 2026 Sep 3;17:1904890. doi: 10.3389/fendo.2026.1904890 (PMC13581627; doi:10.3389/fendo.2026.1904890)
Supplement: Supplementary file 1 [file Table1.docx]

**Supplementary Table 1. Proportional Hazards Assumption Test for the Original Model (Schoenfeld Residuals Method)**

| **Variable** | **chisq** | **df** | **p_value** |
| --- | --- | --- | --- |
| SII_Q4 | 17.677 | 3 | **<0.001** |
| sex_numeric | 1.794 | 1 | 0.1804 |
| Hypertension | 0.234 | 1 | 0.6282 |
| Coronary artery disease | 21.7 | 1 | **<0.001** |
| Dyslipidemia | 0.912 | 1 | 0.3396 |
| Diabetic kidney disease | 0.58 | 1 | 0.4461 |
| Chronic kidney disease | 9.737 | 1 | **0.0018** |
| NAFLD | 4.374 | 1 | **0.0365** |
| GLOBAL | 49.86 | 10 | **<0.001** |

Bold: P < 0.05

**Supplementary Table 2. Proportional Hazards Assumption Test for the Stratified Model (Sensitivity Analysis)**

| **Variable** | **chisq** | **df** | **p_value** |
| --- | --- | --- | --- |
| SII_Q4 | 13.821 | 3 | **0.0032** |
| sex_numeric | 0.177 | 1 | 0.6743 |
| Hypertension | 0.759 | 1 | 0.3836 |
| Dyslipidemia | 0.074 | 1 | 0.7856 |
| Diabetic kidney disease | 0.807 | 1 | 0.3689 |
| GLOBAL | 16.007 | 7 | **0.0251** |

Bold: P < 0.05

**Supplementary Table 3. Sensitivity Analysis 2: Traditional Cox Regression Models (Models 1/2/3)**

| **Model 1** | | |  | **Model 2** | | |  | **Model 3** | | | |
| --- | --- | --- | --- | --- | --- | --- | --- | --- | --- | --- | --- |
| **Variable** | **Group** | **HR (95% CI)** | **P** | **Variable** | **Group** | **HR (95% CI)** | **P** | **Variable** | **Group** | **HR (95% CI)** | **P** |
| Albumin | Q1 | 1.00 (Reference) |  | Albumin | Q1 | 1.00 (Reference) |  | Albumin | Q1 | 1.00 (Reference) |  |
|  | Q2 | 1.13 (0.97-1.31) | 0.1225 |  | Q2 | 1.12 (0.97-1.3) | 0.1269 |  | Q2 | 1.05 (0.9-1.22) | 0.5329 |
|  | Q3 | 1.31 (1.13-1.51) | **<0.001** |  | Q3 | 1.31 (1.13-1.51) | **<0.001** |  | Q3 | 1.19 (1.03-1.37) | **0.0205** |
|  | Q4 | 1.19 (1.03-1.37) | **0.0176** |  | Q4 | 1.17 (1.02-1.35) | **0.0297** |  | Q4 | 1.06 (0.92-1.23) | 0.4053 |
| BMI | Q1 | 1.00 (Reference) |  | BMI | Q1 | 1.00 (Reference) |  | BMI | Q1 | 1.00 (Reference) |  |
|  | Q2 | 0.96 (0.84-1.1) | 0.5899 |  | Q2 | 0.94 (0.82-1.07) | 0.3623 |  | Q2 | 0.89 (0.78-1.02) | 0.0907 |
|  | Q3 | 1.05 (0.92-1.18) | 0.472 |  | Q3 | 1.02 (0.9-1.15) | 0.8103 |  | Q3 | 0.93 (0.82-1.05) | 0.2488 |
|  | Q4 | 1.04 (0.91-1.19) | 0.5269 |  | Q4 | 1.03 (0.9-1.18) | 0.6663 |  | Q4 | 0.93 (0.81-1.06) | 0.2683 |
| MHR | Q1 | 1.00 (Reference) |  | MHR | Q1 | 1.00 (Reference) |  | MHR | Q1 | 1.00 (Reference) |  |
|  | Q2 | 1.11 (0.97-1.27) | 0.1145 |  | Q2 | 1.08 (0.95-1.23) | 0.2457 |  | Q2 | 1.06 (0.93-1.21) | 0.407 |
|  | Q3 | 1.18 (1.03-1.34) | **0.0153** |  | Q3 | 1.13 (0.99-1.29) | 0.0687 |  | Q3 | 1.07 (0.94-1.22) | 0.3071 |
|  | Q4 | 1.28 (1.12-1.46) | **<0.001** |  | Q4 | 1.21 (1.06-1.39) | **0.0058** |  | Q4 | 1.16 (1.02-1.33) | **0.0295** |
| NHR | Q1 | 1.00 (Reference) |  | NHR | Q1 | 1.00 (Reference) |  | NHR | Q1 | 1.00 (Reference) |  |
|  | Q2 | 1.2 (1.05-1.36) | **0.0063** |  | Q2 | 1.17 (1.03-1.33) | **0.0145** |  | Q2 | 1.12 (0.98-1.27) | 0.0851 |
|  | Q3 | 1.21 (1.06-1.37) | **0.0046** |  | Q3 | 1.18 (1.03-1.34) | **0.0143** |  | Q3 | 1.12 (0.98-1.28) | 0.0913 |
|  | Q4 | 1.26 (1.1-1.44) | **<0.001** |  | Q4 | 1.22 (1.06-1.39) | **0.0045** |  | Q4 | 1.16 (1.01-1.32) | **0.0348** |
| NLR | Q1 | 1.00 (Reference) |  | NLR | Q1 | 1.00 (Reference) |  | NLR | Q1 | 1.00 (Reference) |  |
|  | Q2 | 1.09 (0.96-1.22) | 0.1833 |  | Q2 | 1.07 (0.95-1.2) | 0.2903 |  | Q2 | 1.06 (0.94-1.19) | 0.3643 |
|  | Q3 | 1.13 (0.99-1.28) | 0.0618 |  | Q3 | 1.1 (0.97-1.25) | 0.1258 |  | Q3 | 1.11 (0.98-1.26) | 0.0988 |
|  | Q4 | 0.97 (0.84-1.11) | 0.6472 |  | Q4 | 0.95 (0.82-1.09) | 0.4406 |  | Q4 | 0.98 (0.85-1.13) | 0.7618 |
| PLR | Q1 | 1.00 (Reference) |  | PLR | Q1 | 1.00 (Reference) |  | PLR | Q1 | 1.00 (Reference) |  |
|  | Q2 | 1.07 (0.95-1.21) | 0.2828 |  | Q2 | 1.08 (0.95-1.22) | 0.2238 |  | Q2 | 1.06 (0.94-1.19) | 0.3693 |
|  | Q3 | 1.06 (0.94-1.2) | 0.3453 |  | Q3 | 1.08 (0.95-1.22) | 0.2246 |  | Q3 | 1.08 (0.96-1.23) | 0.2143 |
|  | Q4 | 0.9 (0.78-1.03) | 0.1235 |  | Q4 | 0.91 (0.79-1.05) | 0.1894 |  | Q4 | 0.95 (0.82-1.09) | 0.4618 |
| SII | Q1 | 1.00 (Reference) |  | SII | Q1 | 1.00 (Reference) |  | SII | Q1 | 1.00 (Reference) |  |
|  | Q2 | 1.19 (1.05-1.34) | **0.0065** |  | Q2 | 1.18 (1.05-1.34) | **0.0074** |  | Q2 | 1.14 (1.01-1.29) | **0.0394** |
|  | Q3 | 1.2 (1.06-1.37) | **0.0044** |  | Q3 | 1.2 (1.06-1.37) | **0.0041** |  | Q3 | 1.17 (1.03-1.33) | **0.0159** |
|  | Q4 | 1.12 (0.97-1.28) | 0.1135 |  | Q4 | 1.12 (0.97-1.28) | 0.1112 |  | Q4 | 1.12 (0.97-1.28) | 0.1132 |
| SIRI | Q1 | 1.00 (Reference) |  | SIRI | Q1 | 1.00 (Reference) |  | SIRI | Q1 | 1.00 (Reference) |  |
|  | Q2 | 1.04 (0.91-1.17) | 0.5826 |  | Q2 | 1 (0.88-1.14) | 0.9768 |  | Q2 | 0.98 (0.86-1.11) | 0.7214 |
|  | Q3 | 1.21 (1.07-1.37) | **0.0031** |  | Q3 | 1.17 (1.04-1.33) | **0.0124** |  | Q3 | 1.14 (1.01-1.3) | **0.0388** |
|  | Q4 | 1.1 (0.96-1.26) | 0.1603 |  | Q4 | 1.05 (0.92-1.21) | 0.4479 |  | Q4 | 1.08 (0.94-1.24) | 0.3055 |
| Uric acid | Q1 | 1.00 (Reference) |  | Uric acid | Q1 | 1.00 (Reference) |  | Uric acid | Q1 | 1.00 (Reference) |  |
|  | Q2 | 1.23 (1.07-1.42) | **0.0046** |  | Q2 | 1.2 (1.04-1.38) | **0.0141** |  | Q2 | 1.12 (0.97-1.3) | 0.1109 |
|  | Q3 | 1.31 (1.13-1.5) | **<0.001** |  | Q3 | 1.24 (1.08-1.43) | **0.0029** |  | Q3 | 1.15 (0.99-1.32) | 0.0633 |
|  | Q4 | 1.17 (1.02-1.34) | **0.0279** |  | Q4 | 1.11 (0.97-1.28) | 0.1244 |  | Q4 | 1 (0.87-1.15) | 0.9812 |
| eGFR | Q1 | 1.00 (Reference) |  | eGFR | Q1 | 1.00 (Reference) |  | eGFR | Q1 | 1.00 (Reference) |  |
|  | Q2 | 1.06 (0.93-1.21) | 0.4009 |  | Q2 | 1.07 (0.93-1.22) | 0.3457 |  | Q2 | 1.03 (0.9-1.19) | 0.6258 |
|  | Q3 | 0.97 (0.85-1.11) | 0.6995 |  | Q3 | 0.97 (0.85-1.11) | 0.6753 |  | Q3 | 0.97 (0.85-1.11) | 0.6912 |
|  | Q4 | 0.92 (0.81-1.05) | 0.239 |  | Q4 | 0.91 (0.8-1.04) | 0.1798 |  | Q4 | 0.94 (0.82-1.08) | 0.4041 |

HR: Hazard Ratio, CI: Confidence Interval
Model 1: Crude (unadjusted)
Model 2: Adjusted for Sex,

Model 3: Fully adjusted (Sex + Hypertension + CAD + Dyslipidemia + DKD + CKD + NAFLD)

Bold: P < 0.05
